# Psychophysiological techniques and energy medicine

**DOI:** 10.4103/0973-6131.78184

**Published:** 2011

**Authors:** Satendra Singh

**Affiliations:** University College of Medical Sciences, Dilshad Garden, Delhi, India

Sir,

The editorial on ‘Energy medicine’ briefly introduced this topic in the last issue. Energy medicine is being more repeatedly used in sustaining patients to attain best possible health. The concept of energy medicine as a holistic practice was introduced, and its relationship to physical medicine discussed in 2004.[1] Many proponents of complementary and alternative medicine assume the existence of a vital force that mediates therapeutic efficacy, for example chi or qi in traditional Chinese medicine. Vital energy directly perceived or imaged that surrounds living organisms is frequently termed the aura. A range of psychophysiological techniques are claimed to image this vital energy or aura. These include Kirlian photography, gas discharge visualization (GDV) and polychromatic interference photography (PIP).

Their use as diagnostic or imaging alternatives may be clinically debatable but as a as a research tool they provide responses to physiological or psychological stressors.[2] These methods assess electro-optical parameters of the skin based on the registration of physical processes emerging from electron components of tissue conductivity.[3] Kirlian radiation was found to be useful for early detection of disturbances in energy circulation of brain vessels.[4] However, there is disagreement among researchers who think that there are simple, scientific explanations for these paranormal Kirlian effects.[5]

A recent study explored the alteration of the sympathetic nervous system in autistic children using the GDV.[6] They found that the differences between autistic children and controls expressed on the psycho-emotional level were the most significant as compared to the other groups.

These unconventional therapies in environmentalmedicine are not without their share of criticism. Better structured randomized controlled trials will go a long way in providing legitimacy to these psychophysiological techniques. The *International Journal of Yoga* can provide the platform for evidencebased research in this regard.
